# Hypothyroidism: The difficulty in attributing symptoms to their underlying cause

**DOI:** 10.3389/fendo.2023.1130661

**Published:** 2023-02-06

**Authors:** Heleen I. Jansen, Anita Boelen, Annemieke C. Heijboer, Eveline Bruinstroop, Eric Fliers

**Affiliations:** ^1^ Department of Clinical Chemistry, Endocrine Laboratory, Amsterdam University Medical Centers (UMC) Vrije Universiteit Amsterdam, Amsterdam, Netherlands; ^2^ Amsterdam Gastroenterology, Endocrinology & Metabolism, Amsterdam University Medical Centers (UMC), Amsterdam, Netherlands; ^3^ Department of Clinical Chemistry, Endocrine Laboratory, Amsterdam University Medical (UMC) Centers, University of Amsterdam, Amsterdam, Netherlands; ^4^ Amsterdam Reproduction & Development Research Institute, Amsterdam University Medical Centers (UMC), Amsterdam, Netherlands; ^5^ Department of Endocrinology & Metabolism, Amsterdam University Medical Centers (UMC), University of Amsterdam, Amsterdam, Netherlands

**Keywords:** subclinical hypothyroidism, thyroid disease-related symptoms, levothyroxine, liothyronine, patient-reported outcomes (PROs)

## Abstract

Common symptoms of overt hypothyroidism are non-specific and include fatigue, lethargy, and dry skin. Although the diagnosis is considered to be straightforward, no single symptom can be used to identify patients with overt hypothyroidism, while many patients with subclinical hypothyroidism are asymptomatic. A large population-based study on the spectrum of symptoms in subclinical hypothyroidism showed similar rates of thyroid disease-related symptoms compared with euthyroid subjects, while the TSH concentration had no impact on symptom score. Together, these findings make it challenging to attribute symptoms to their underlying cause. This is also true in the case of unexplained persistent symptoms in levothyroxine-treated patients. Although generally considered a life-long replacement therapy, successful thyroid hormone discontinuation resulting in euthyroidism has been reported in approximately one third of patients. Thus, we overtreat patients with (subclinical) hypothyroidism, highlighting the importance of reliable diagnostic criteria. The diagnostic process, including the implementation of robust TSH and FT4 reference intervals, is especially challenging in specific situations including aging, pregnancy, non-thyroidal illness, and central hypothyroidism. There is a clear need for improved adherence to current guidelines from scientific societies and for willingness to manage symptoms without a clear pathological correlate, especially in the case of mild TSH elevations. This review will highlight recent literature on this topic and offers some practice points.

## Introduction

Hypothyroidism is a condition with an estimated prevalence in the general population of 0.3% - 3.7% in the USA and 0.2%-5.3% in Europe, depending on the definition used (1). Approximately 12% of the adult population has subclinical hypothyroidism (2, 3). The diagnosis and treatment of (subclinical) hypothyroidism has traditionally been considered rather straightforward. On closer examination, however, many unresolved issues remain and patient satisfaction in terms of symptom relief is often suboptimal (4). Several factors may contribute to this situation. First, common symptoms of overt hypothyroidism including weight gain and fatigue are non-specific and may result from factors other than a shortage of thyroid hormone. In this situation, thyroid hormone replacement can be expected to be less effective in terms of restoring well-being than anticipated. Second, persons with subclinical hypothyroidism experience thyroid disease-related symptoms as often as euthyroid subjects as shown in a recent study. This may explain why symptomatic relief following levothyroxine (L-T4) substitution in this patient group is often disappointing (5). Third, over the past decades it has become clear that the impact of thyroid disease on quality of life is an important disease aspect that is best investigated by patient-reported outcomes (PROs) such as the thyroid-related quality of life patient-reported outcome measure (ThyPRO) (6), whereas ‘classic’ symptoms of hypothyroidism may be less reliable to assess thyroid hormone status. The impact of primary hypothyroidism on quality of life has recently been reviewed by Hegedüs et al. (7).

Thus, it is not surprising that PROs are increasingly used in clinical practice with the aim to improve clinical care. Systematic use of PROs can enhance communication with patients, thereby improving patient satisfaction, symptom management and quality of life. For an overview of the challenges to be handled to achieve successful implementation of PROs in routine clinical practice see the minireview by Cramon et al. in the present article collection on “(Re)defining hypothyroidism: the key to patient-centered treatment” (8). A final factor that may contribute to less than optimal patient management and symptom relief in hypothyroidism is the pivotal role of plasma thyroid stimulating hormone (TSH) and free thyroxine (FT4) both in diagnosis and treatment. Health care providers rely exclusively on elevated plasma TSH and decreased FT4 concentrations as key biochemical criteria for the diagnosis of overt primary hypothyroidism, while subclinical hypothyroidism is defined as an elevated TSH with a FT4 concentration within the reference interval. This illustrates the importance of the availability of robust methods and validated TSH and FT4 reference intervals. Another complex aspect of biochemical monitoring is uncertainty about the optimal target range of TSH and FT4 during thyroid hormone replacement. Moreover, it is questionable whether plasma TSH and FT4 concentrations reliably reflect thyroid hormone status at the tissue level. Finally, the biochemical diagnosis may be especially challenging in specific situations including pregnancy, non-thyroidal illness, aging, and central hypothyroidism. In the present brief review we will discuss the above mentioned issues in more detail, highlighting recent literature on this topic and addressing some practice points.

## Interpretation of symptoms in diagnosing and treating overt hypothyroidism

Overt hypothyroidism is defined as a high TSH combined with a low FT4 concentration. Common symptoms of hypothyroidism in adults include fatigue, lethargy, cold intolerance, weight gain, constipation, and dry skin, while the clinical presentation shows large intra-individual variation depending on age, sex, and time interval between onset and diagnosis (9). Importantly, these symptoms for the diagnosis of hypothyroidism are non-specific. In a population based case-control study hypothyroid patients suffered mostly from tiredness (81%), dry skin (63%), and shortness of breath (51%). Among 34 symptoms investigated, only 13 symptoms were statistically overrepresented in hypothyroidism. None of 34 hypothyroidism-related symptoms could be used to identify patients with hypothyroidism (10). Together, these findings make it challenging to attribute symptoms to their underlying cause.

However, when overt hypothyroidism is left untreated it is known to be associated with implications for most organs, including the cardiovascular system. It may result in increased vascular resistance, decreased cardiac output and left ventricular function, and changes in several other markers of cardiovascular contractility (1). A detailed study showed diffuse myocardial injuries to be common in patients with hypothyroidism (11). The combination of non-specific symptoms of hypothyroidism, potentially harmful effects of overt hypothyroidism for vital organ function and the availability of a cheap and reliable diagnostic TSH test has paved the way for a policy that even minor suspicion should lead to a blood test. This may have contributed to the current situation where L-T4 is one of the most commonly prescribed medications and it is even prescribed if thyroid function tests are normal (12). Guidelines advise treatment of all patients with a combination of high TSH and low FT4 concentrations irrespective of the presence of symptoms. Although generally considered a life-long replacement therapy, successful discontinuation has been reported. Burgos et al. (13) performed a systematic review and meta-analysis of clinical outcomes after discontinuation of thyroid hormone replacement including a total of 1103 patients. Surprisingly, approximately a third of patients undergoing thyroid hormone discontinuation remained euthyroid at follow-up. Patients with a previous diagnosis of overt hypothyroidism were less likely to remain euthyroid (11.8%) than patients with a prior diagnosis of subclinical hypothyroidism (35.6%). Thus, to prevent overtreatment, a systematic management procedure may be warranted to help clinicians reevaluate the need for levothyroxine in their patients.

## Interpretation of symptoms in diagnosing and treating subclinical hypothyroidism

Many patients with subclinical hypothyroidism are asymptomatic, but others report symptoms of overt hypothyroidism more often than euthyroid controls; these symptoms are usually milder than those in patients with overt hypothyroidism (5, 14, 15). Some, but not all, studies have shown increased rates of depressive symptoms, reduced quality of life, cognitive function, and memory among persons with subclinical hypothyroidism (16–18), while other studies reported fatigue, muscle weakness, weight gain, cold intolerance, and constipation (19). Some of these studies had a rather small sample size or focused on patients with a limited TSH range. As plasma TSH will normalize, without treatment, in up to 46% of patients with subclinical hypothyroidism and mildly elevated TSH, a transient increase in TSH should be ruled out by a repeat measurement after 2 to 3 months (20). In addition to subclinical hypothyroidism resulting from mild thyroid disease, TSH may be slightly elevated as result of the circadian TSH rhythm, obesity, or rare mutations in the TSH receptor that do not always necessitate levothyroxine treatment (21, 22).

In a large population-based Danish study on the spectrum of symptoms in subclinical hypothyroidism, patients with a thyroid function test suggesting subclinical hypothyroidism did not experience thyroid disease-related symptoms more often than euthyroid subjects. Of note, the measured TSH concentration had no impact on symptom score. The largest difference in symptoms was between patients with and without co-morbidities. The authors concluded that clinicians should focus on concomitant diseases in patients with subclinical hypothyroidism rather than expecting symptomatic relief following L-T4 substitution (5). In line with these observations, a prospectively planned analysis of data from 2 clinical trials involving adults over 80 years of age with subclinical hypothyroidism, treatment with L-T4 was not significantly associated with improvement in hypothyroid symptoms or fatigue compared with placebo. These observations plead against routine use of L-T4 for treatment of subclinical hypothyroidism in this age group (23). On the other hand, a recent study by Shao et al. (24) showed that the metabolic profile of overt hypothyroid patients resembled the metabolic profile of patients with subclinical hypothyroidism. This suggests that some patients with subclinical hypothyroidism may display a similar risk profile to develop organ damage due to untreated thyroid disease as overt hypothyroid patients and may benefit from L-T4 treatment. However, it is not well-established yet how this metabolic profile behaves during L-T4 treatment. Furthermore, this study included participants between 18 and 60 years and may not be representative for the older adult population.

Based on the clinical studies mentioned, some controversy has arisen regarding the treatment of subclinical hypothyroidism. Current ATA and ETA guidelines advise to treat subclinical hypothyroidism with L-T4 if TSH is ≥10 mIU/L or if TSH ≥ 4-4.5 mIU/L in combination with symptoms and/or comorbidities (25, 26), while also taking age into account. A TSH concentration ≥ 10 mIU/L seemed to be related with adverse cardiovascular outcomes and increased mortality (27). However, a more recent guideline issued a recommendation against thyroid hormone treatment in subclinical hypothyroidism (28). In a debate article, Peeters and Brito (29) noted that the care for patients with subclinical hypothyroidism and mild symptoms remains a dilemma. On the one hand, failure to act and correct the laboratory abnormality may be perceived by the patient as uncaring, but then again the same may be true for acting on a laboratory test without assisting the patient and relieving symptoms. In order to stop a further rise in unnecessary L-T4 prescriptions, the same authors advocated improved adherence to current guidelines from scientific societies and stressed the need for clinicians to become more expert in the management of symptoms without a clear pathological correlate, especially in the case of mild TSH elevations (29). In the absence of a full understanding of the pathophysiology and meaning of mild symptoms in patients with subclinical hypothyroidism, patient and clinician may choose to conduct a 6-month therapeutic trial with L-T4. Symptoms should be reassessed periodically and treatment discontinued if and when no benefit becomes evident (26).

## Persistent symptoms in levothyroxine-treated patients

Despite TSH concentrations within the reference interval, 5-10% of hypothyroid patients receiving L-T4 report impaired psychological well-being, depression or anxiety (30). Furthermore, the presence of so-called brain fog in L-T4 treated hypothyroid patients is a well-known phenomenon experienced by many patients (31). Several mechanisms may underlie the discrepancy between ‘normal’ laboratory outcomes and persistent symptoms. Over the last decades, many studies focused on the possible role of low triiodothyronine (T3) during L-T4 treatment. The assumption is that L-T4 substitution alone does not suffice, as it leads to low/low-normal (F)T3 and high FT4 concentrations in the presence of TSH concentrations within the reference interval (32). Furthermore, a recent study analyzing the relationships of FT4, T3 and TSH concentrations in L-T4 treated patients showed that L-T4 dose changes that robustly modified serum FT4 and TSH concentrations affected serum T3 concentrations only minimally (33). Several trials investigated the added value of combination treatment of L-T4 and L-T3 and until now, no clear benefit of L-T3 was found (34). However, these studies used different L-T4/T3 ratios, were sometimes underpowered, and used a variety of primary endpoints. A recent consensus statement was published to guide the development of future clinical trials of L-T4/L-T3 combination therapy. The results of such redesigned trials can be expected to be of benefit to patients and to help develop future thyroid hormone replacement treatment recommendations (34).

A recent review article (35) discussing persistent symptoms in L-T4 treated patients emphasized the importance and underexposure of alternative explanations besides the low T3 theory. For example, persistent symptoms might be due to the incidental coexistence of treated hypothyroidism with a diagnosis of Somatic Symptom and Related Disorders (SSRD). The term SSRD refers to the persistence of physical symptoms along with physiological problems, large functional impairment and high healthcare costs. Furthermore, additional effects of autoimmunity may play a role. At first sight, this theory was supported by a study showing that L-T4 treated patients with continuously high TPO-antibodies had more often persistent symptoms, which improved spectacularly after thyroidectomy. However, there was no control group for the surgical intervention (36). A recent meta-analysis failed to detect an association between TPO-antibodies and persistent symptoms, although a link between TPO-antibodies and decreased quality of life was suggested (37). Moreover, (undetected) autoimmune comorbidities, chronic medication use, lifestyle, and unrealistic expectations may play a role in persistence of symptoms as well.

Finally, plasma TSH and FT4 concentrations may not fully represent tissue thyroid hormone (TH) concentrations during treatment since TH bioavailability may differ per target organ. This could lead to a mismatch in plasma TH concentrations and symptoms in L-T4 treated patients. In the present article collection on “(Re)defining hypothyroidism: the key to patient-centered treatment”, Salas & Bianco (38) advocated the use of (free) triiodothyronine ((F)T3) during treatment, as kinetic studies revealed that plasma (F)T3 levels can accurately predict tissue T3 content and T3 signaling in most tissues, except for the brain. Given this direct relationship between plasma and tissue T3 contents and T3 signaling in most tissues, the authors suggest that clinicians managing patients with hypothyroidism should refocus attention on monitoring plasma (F)T3 levels in addition to TSH. Although many symptoms of hypothyroidism, including persistent symptoms during treatment, have their origin in the brain it is still challenging to assess brain TH status. One study using functional MRI in a group of long-term L-T4 treated and euthyroid patients reported no cognitive or neural alterations, while current mood status could not be related to depression-related networks (39). However, since autoimmune activity and treatment duration did show a relationship with depression and hypothyroidism-related brain structure and function, the authors concluded that more research using functional brain imaging is needed to replicate and enhance the research effort on residual complaints despite biochemically adequate treatment in patients with Hashimoto’s thyroiditis. More and larger studies are lacking and demand for these studies is increasing.

## Symptoms after brand switch

Besides persistent thyroid disease-related symptoms during L-T4 therapy, many patients report similar symptoms upon L-T4 brand switch. A recent study examined the effect of the shortage of the L-T4 brand Thyrax^®^ in the Netherlands and the resulting dose-equivalent switch to another brand on plasma TSH concentrations in a large cohort of patients. First, the number of reported symptoms increased according to the Netherlands Pharmacovigilance Centre Lareb. Using data from the Nivel Primary Care Database and the PHARMO Database Network it was shown that in euthyroid patients continuing the L-T4 product Thyrax at the same dose, TSH was out of range in +/-20% >6 weeks later. Of note, a dose-equivalent switch from Thyrax to other L-T4 brands induced biochemical signs of overdosing in a much larger proportion (24-63%) of patients. Thus, a dose-equivalent L-T4 brand switch may necessitate a dose adjustment in a large number of patients (40). Current recommendations do not support an unnecessary switch of L-T4 brands, and advise, if a switch is necessary, to evaluate TSH concentrations after 6 weeks (41, 42). It should be noted that the outcome of a recent study by Brito et al. (43) was different, as it did not show a change in serum TSH concentrations after L-T4 brand switch. Thus, it is likely that the effect of a L-T4 brand switch on TSH depends on the particular brand change. As both the studies by Brito et al. and Flinterman et al. did not take thyroid disease-related symptoms into account, it is not clear whether increased symptoms and altered TSH after brand change are causally related. Thus, more research is warranted before definite conclusions can be drawn.

## Pitfalls of biochemical measurements in primary (subclinical) hypothyroidism

As discussed, laboratory measurements of TSH and FT4 currently play a key role to diagnose primary hypothyroidism and monitor L-T4 treatment. In case of suspected primary hypothyroidism, assessment of plasma TSH suffices and is considered to provide enough insight into TH status. To differentiate between overt primary hypothyroidism and subclinical hypothyroidism, addition of the plasma FT4 concentration is necessary and the concentrations must be below the lower reference interval to confirm overt hypothyroidism. Current ATA and ETA guidelines advise to treat subclinical hypothyroidism with L-T4 if TSH is ≥10 mIU/L or if TSH ≥ 4-4.5 mIU/L in combination with symptoms and/or comorbidities (25, 26), while also taking age into account. As discussed already, the assessment and interpretation of symptoms can be difficult. Thus, laboratory measurements should be robust since they form the primary basis for treatment decisions, and reference intervals should be correctly interpreted.

TSH and FT4 concentrations are mostly measured using automated immunoassays (IAs) in clinical laboratories. It is important to realize that these hormones are measured using IAs produced by various manufacturers (e.g., Roche, Siemens, and Abbott). These IAs are not standardized and therefore have different reference intervals meaning that results obtained from an IA of one manufacturer cannot be directly compared to the results derived from an IA of another manufacturer (44, 45). Therefore, it is remarkable that many guidelines mention absolute TSH cut-off values without specifying the IAs, since TSH concentrations can vary depending on the measurement method that is used. Although the step towards harmonization of TSH measurements has been taken by the IFCC Committee for Standardization of Thyroid Function Tests, this has not been implemented yet and caution is required in extrapolating cut-off values to different laboratories with different IAs (44). The same is true for the interpretation of FT4 concentrations. Even though more attention is paid to differences in FT4 reference intervals between different IAs, awareness is still warranted. Moreover, most FT4 IAs perform well in large healthy cohorts but show difficulties in several groups characterized by a different blood composition such as pregnant women and patients with advanced renal failure (46, 47).

Another pitfall that should be acknowledged is that not all groups benefit from using the same TSH and FT4 reference intervals. Clinical studies in euthyroid patients using L-T4 showed that FT4 concentrations in this group are significantly higher, and more often above the upper reference interval, than in non L-T4 treated people (32, 48, 49) indicating that in this group a higher upper reference limit may apply. In addition, FT3 concentrations and FT3/FT4 ratios are lower in this group compared to non-L-T4 users, a finding probably more outspoken in athyreotic patients due to the absence of any endogenous T3 production. Therefore, the question is justified whether the current reference intervals suffice for persons using L-T4. As mentioned previously, Salas & Bianco (38) suggested that (F)T3 concentrations may reflect TH status in L-T4 treated people even better than FT4. On the other hand, an analysis of the relationship between psychological well-being and FT4, FT3 and TSH in a large group of patients on L-T4 showed that psychological well-being correlates with FT4 but not with FT3 levels (50). Thus, optimal biochemical monitoring during TH treatment is still a matter of debate and pitfalls regarding the measurement and interpretation of TSH and FT4 should be acknowledged and taken into account by laboratory specialists and clinicians.

## Primary hypothyroidism in specific groups

Diagnosis and monitoring of primary (subclinical) hypothyroidism in specific (patient) groups can cause several dilemmas since these groups are not directly comparable to healthy cohorts. Both symptoms and laboratory measurements may present differently. Several of these groups are discussed.

### Aging

Both symptoms of hypothyroidism and interpretation of laboratory results are different in the older adult population. First, older adults with subclinical hypothyroidism have fewer symptoms than younger persons with subclinical hypothyroidism (51–54). Furthermore, treatment of subclinical hypothyroidism with L-T4 in older adults seems to have no positive effect on quality of life and secondary outcomes (55–58). Both aspects complicate the diagnosis and treatment of subclinical hypothyroidism in this group. Second, reference intervals of TSH appear to be different in older adults as TSH concentrations increase somewhat with age (59, 60). This observation may be a physiological manifestation of aging indicating the need for age-specific reference intervals. However, their practical applicability is still under debate since an age-related TSH increase usually does not exceed the advised cut-off concentrations for treatment (61). Current guidelines for subclinical hypothyroidism do take age into account, and starting L-T4 treatment below a TSH concentration of 10 mIU/L in persons >70 years of age is not recommended (26, 62). In our opinion, subclinical hypothyroidism and its treatment in older adults should probably be a matter of personalized medicine and shared decision making. In case of overt primary hypothyroidism, on the other hand, L-T4 treatment is usually advised since overall mortality is significantly higher in hypothyroid compared to euthyroid older adults (63). However, in community-dwelling people aged 80 years and older, (sub-)clinical thyroid dysfunction was not associated with functional outcomes or mortality and may therefore be of limited clinical significance (64). Another study even showed a lower risk for nursing home admission and all-cause mortality with increasing TSH in people over 80 years old (65). The ATA guidelines from 2012 advised to use an adjusted TSH upper limit in older adults, although a specific age was not mentioned nor the indications for treatment (25).

### Pregnancy

Overt hypothyroidism and subclinical hypothyroidism during pregnancy can be challenging for several reasons. First, hypothyroid symptoms during pregnancy show large overlap with symptoms of pregnancy itself and symptoms presented in the general population (66, 67) which makes it difficult to fully rely on these symptoms. Furthermore, hypothyroid symptoms were not related to TSH concentrations (68). Since it is not possible to fully rely on symptoms, it would be ideal to depend on TSH and FT4 concentrations. This brings us to the second challenge, since TSH and FT4 concentrations change during pregnancy. Generally, TSH concentrations decrease slightly during the first trimester and increase during the rest of pregnancy, while FT4 concentrations first increase and subsequently decrease from the second trimester. This results in different reference intervals compared to the non-pregnant situation (69). The observed change is considered physiological, although the exact mechanism is still unclear. A recent study showed that immunoassays overestimate FT4 concentrations in pregnant women while the degree of overestimation differs between several frequently used immunoassays (47). Therefore, it is recommended not only to use pregnancy-specific reference intervals for TSH and FT4 (69), but ideally these reference intervals should be trimester- and immunoassay-specific as well. However, this is not the standard situation in clinical laboratories yet, which complicates the diagnosis and adequate treatment of hypothyroidism in pregnant women. The ATA guideline recommended to subtract 0.5 mIU/L from the lower reference limit of TSH during pregnancy, a recommendation that was recently implemented in the Dutch guideline as well (70, 71). The ETA, on the other hand, advises to either establish trimester-specific reference intervals per hospital or to use the following upper limits for TSH: first trimester, 2.5 mU/l; second trimester, 3.0 mU/l; third trimester, 3.5 mU/l (72). Finally, the optimal treatment strategy in pregnant women with subclinical hypothyroidism is debated and controversy still remains. It seems particularly important to reach multidisciplinary consensus on this topic (73).

### Non-thyroidal illness syndrome

Diagnosing and treating thyroid dysfunction in severely ill patients is often a challenge. The non-thyroidal illness syndrome (NTIS), with decreased FT3 and paradoxically low TSH in patients without any thyroid pathology, was first reported in the 1970s as a remarkable ensemble of changes in plasma TH concentrations occurring in probably any severe illness. Ever since, NTIS has remained an intriguing phenomenon not only because of the robustness of the decrease in plasma FT3, but also by its clear correlation with morbidity and mortality (74). NTIS can be difficult to diagnose and especially the distinction between severe primary hypothyroidism and NTIS may be difficult (75). NTIS is characterized by decreased FT3 concentrations, and in severe illness decreased FT4 concentrations, without a concomitant rise in plasma TSH concentrations. Substitution treatment with L-T4 is not necessary. An increased TSH in critically ill patients, on the other hand, is indicative of primary hypothyroidism and should lead to treatment with L-T4. Unfortunately, an absence of clearly increased TSH is not enough for ruling out primary hypothyroidism. In this setting, the combination of a high FT3/FT4 ratio and a low reversed T3 (rT3) concentration points to primary hypothyroidism. These measurements are currently not widely available, indicating challenges still remain in differentiating between NTIS and primary hypothyroidism in critically ill patients. Finally, tissue TH concentrations during NTIS can differ substantially between several organs within and between patients even in the presence of similar TSH, FT4 and FT3 concentrations in plasma (74).

### Central hypothyroidism

Central hypothyroidism (CeH) is a rare form of hypothyroidism due to disturbances at the level of the hypothalamus or pituitary resulting in insufficient stimulation of the thyroid gland. The pathogenesis of CeH is variable and can be either congenital or acquired (e.g. iatrogenic, or by trauma, or autoimmunity). Furthermore, CeH can be isolated or part of a multiple pituitary hormone deficiency (MPHD). Due to the several etiologies, the clinical presentation of CeH is heterogeneous and does not only involve classical hypothyroid symptoms. In patients with MPHD, the clinical presentation depends on its etiology, and may reflect, e.g., a pituitary adenoma, or a history of craniospinal irradiation in combination with symptoms caused by endocrine deficiencies in several endocrine axes. Recently, mutations of *IGSF1, TBL1X* and *IRS4* leading to CeH were detected in addition to the mutations in *TRH-R* and *TSHβ* reported earlier (76–80). A *IGSF1* mutation leading to CeH is also characterized by macroorchidism and the *TBL1X* mutation by impaired hearing. These discriminative features can be used for guidance into the cause of CeH.

Since central control of the thyroid gland is lacking in CeH, TSH concentrations are low/normal and do not reflect TH status adequately in those patients. Therefore, FT4 concentrations are key in diagnosing and monitoring CeH. In most newborn screening programs for congenital hypothyroidism, TSH is measured as a first step. Since CeH is most often characterized by a low/normal TSH concentration, it can be challenging to detect CeH with this strategy. Furthermore, mild forms of CeH might even present with a FT4 concentration around the lower border of the reference interval and are therefore even more challenging to detect. Congenital CeH can be reliably detected in the newborn screening only if the protocol measures T4 as a first tier. While this is the case in The Netherlands and Japan, a TSH-based newborn screening is far more common and will not diagnose newborns with CeH.

The cornerstone of treatment of CeH is generally L-T4, although it is important to individually assess treatment necessity. In congenital CeH, children should be treated with L-T4 as soon as possible to ensure optimal neurodevelopment. In milder forms of (congenital) CeH on the other hand, L-T4 treatment must be carefully considered and might be unnecessary, e.g., in older adults with only marginally decreased FT4 concentration based on a mutation in *TBL1X*. If L-T4 is started, low dosage is recommended to avoid risk of overtreatment. TSH cannot reliably be used to monitor L-T4 treatment efficacy and most information about TH status relies on FT4 determination. FT4 concentrations are influenced by the moment of L-T4 intake. Therefore, the European Thyroid Association (ETA) guideline on the diagnosis and management of CeH strongly advises to draw blood before or at least four hours after L-T4 intake (81) to allow reliable results. As previously discussed, FT4 concentrations are often higher in persons on L-T4, leading to the recommendation to pursue FT4 concentrations in the upper range of the reference interval in CeH. FT4 concentrations at the lower range of the reference interval in combination with hypothyroid symptoms indicate under-treatment.

## Conclusion

In this review we discussed the challenges in relating symptoms to biochemical diagnosis and treatment of (subclinical) hypothyroidism. Thyroid related symptoms are mainly non-specific and occur also in the euthyroid population. After treatment with L-T4, many patients report suboptimal symptom relief. There are several hypothetical causes for this phenomenon. The discrepancy between patient satisfaction and biochemical thyroid hormone status may be partly due to the pivotal role of plasma TSH and FT4 concentrations, that do not necessarily reflect tissue thyroid hormone status. Furthermore, symptoms may result from additional factors, including auto-immunity, or having a chronic disorder needing medical attention. Specific situations, such as pregnancy, aging, NTIS, and central hypothyroidism represent extra challenges regarding measurements and interpretation of TSH and FT4 concentrations and monitoring during treatment. There is reason to expect that a) the coordinated design of future clinical trials, b) the further development of innovative monitoring tools including (F)T3 and functional brain imaging, c) the systematic use of PROs to enhance communication with patients, and d) better adherence to international guidelines will lead to an improvement in the expectation and satisfaction of patients with hypothyroidism and of their health care providers.

## Author contributions

HJ and EF participated in the planning process, wrote the manuscript draft, and approved the final manuscript. AB, AH and EB participated in the planning process, read, and commented the manuscript draft, and approved the final manuscript. All authors contributed to the article and approved the submitted version.
